# Investigating the expressions of miRNA-125b and *TP53* in endometriosis. Does it underlie cancer-like features of endometriosis? A case-control study

**DOI:** 10.18502/ijrm.v13i10.7767

**Published:** 2020-10-13

**Authors:** Elnaz Hajimaqsoudi, Farzaneh Darbeheshti, Seyed Mehdi Kalantar, Atiyeh Javaheri, Seyed Hamidreza Mirabutalebi, Mohammad Hasan Sheikhha

**Affiliations:** ^1^Department of Genetics, Faculty of Medicine, Shahid Sadoughi University of Medical Sciences, Yazd, Iran.; ^2^Department of Medical Genetics, School of Medicine, Tehran University of Medical Sciences, Tehran, Iran.; ^3^Breast Cancer Association (BrCA), Universal Scientific Education and Research Network (USERN), Tehran, Iran.; ^4^Abortion Research Center, Yazd Reproductive Sciences Institute, Shahid Sadoughi University of Medical Sciences, Yazd, Iran.; ^5^Department of Obstetrics and Gynecology, Faculty of Medicine, Shahid Sadoughi Hospital, Shahid Sadoughi University of Medical Sciences, Yazd, Iran.; ^6^Student Research Committee, Shahid Sadoughi University of Medical Sciences, Yazd, Iran.

**Keywords:** Endometriosis, TP53, miR-125b, Ectopic endometrium, Eutopic endometrium.

## Abstract

**Background:**

Endometriosis is generally considered as a benign condition; however, there is a possibility for it to become cancerous. miR-125b is upregulated in both endometriotic tissues and serum samples of women with endometriosis but its potential targets in endometriosis are still not fully understood.

**Objective:**

The role of miR-125b in the regulation of *TP53* expression in endometriosis was tested with a bioinformatics approach. In addition, the expression of miR-125b and *TP53* in both eutopic (Eu-p) and ectopic endometrium (Ec-p) in the endometrium tissues of women with endometriosis was compared to those in the normal endometrium tissues of controls (Normal).

**Materials and Methods:**

In this case-control study, the Eu-p and Ec-p samples were collected from 20 women who underwent laparoscopic surgery, and the normal endometrium tissues were collected from 20 controls with no evidence of endometriosis. For bioinformatics approach, a protein-protein interaction network was constructed based on co-expressed potential targets of miR-125b. Quantitative polymerase chain reaction technique was used for the measurement of miR125b and *TP53* expression.

**Results:**

Our results showed that miR-125b was significantly overexpressed in Ec-p (p-value: 0.021). In addition, there was a significant *TP53* under expression in both the Ec-p and Eu-p samples compared with the Normal tissues (p-value: 0.003).

**Conclusion:**

The negative correlation between miR-125b and *TP53* as well as a noticeable decreased expression of *TP53* in both Ec-p and Eu-p samples may be interpreted as the roles of miR-125b/*TP53* axis in the pathogenesis of endometriosis. In addition, these findings and bioinformatic analyses imply a possible role of miR-125b in cancer-like features of endometriosis.

## 1. Introduction 

Endometriosis is a common estrogen-dependent benign disease, characterized by pelvic pain, dysmenorrhea, and infertility (1). It occurs when endometrial tissue exists outside the uterus, and affects 10-15% of women in the reproductive age (2). Although the underlying pathogenesis of endometriosis is still not fully understood, the available body of evidence indicate that both genetics and environmental factors contribute to the susceptibility and progression of endometriosis. While endometriosis is considered as a benign disorder, some cases may represent risk factors for developing estrogen-related malignancies such as ovarian, endometrial, and breast cancers (3-5). However, other types of endometriosis-associated cancers have been rarely reported (6, 7).

Molecular studies have shown a large number of dysregulated transcripts (both coding and noncoding) in the ectopic (Ec-p) and eutopic (Eu-p) endometrial samples from the women with endometriosis (8-11). miRNAs are highly conserved 21-nucleotide single-stranded noncoding RNAs that can bind to target mRNAs and regulate their expression (12). These small noncoding RNAs have a regulatory function in various cellular processes such as proliferation, migration, cell cycle, and apoptosis (13-15). Remarkable dysregulation of miRNAs in diseases results in using them as biomarkers for early diagnosis (16). In addition, miRNAs and their targets can be used for therapeutic purposes (17).

The dysregulation of miR-125 family, as either repressors or promoters, has been seen in several diseases (18). Moreover, a significant upregulation of miR-125b has been found in endometriosis and different cancers (19). In one study, mir-125b in serum samples of women with endometriosis showed more than 10-fold upregulation compared with women without endometriosis (20). It has been suggested that mir-125b plays pivotal role in molecular pathways driving cell proliferation and migration that are the crucial steps for initiating endometriosis and related malignancies (21, 22).

**Figure 1:**
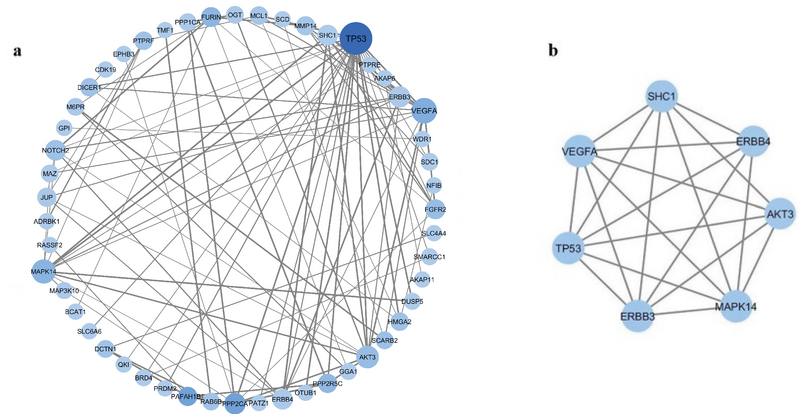
(a) Protein-Protein Interaction (PPI) network of predicted miR-125b targets. The size of node is proportional to the node degree; the node color indicates the betweenness centrality; the width of the edge is proportional to the combined score that has been obtained from STRING database. (b) The top module from protein-protein interaction network.

In this study, the co-expression meta-analysis of miRNA targets (CoMeTa) database was used to identify the potential miR-125b targets. After that, the protein-protein interaction (PPI) network of co-expressed miR-125b targets revealed *TP53* gene as a hub gene, and showed its involvement in the functional module. These pieces of evidence clearly suggest that among potential miR-125b targets, *TP53* has noticeable roles in cellular processes (Figure 1). It was reported that miR-125b caused downregulation of *TP53* by binding to a microRNA element in the 3' untranslated region of *TP53* mRNA (23). For example, it was shown that the overexpression of miR-125b in human neuroblastoma suppresses apoptosis by downregulation of *TP53*. However, in the contrary, reducing the miR-125b level in human lung fibroblasts causes overexpression of *TP53* which in turn induces apoptosis (23).

The most prominent roles of *TP53* are cell-cycle and apoptosis regulation. Previous studies have shown dysregulation of *TP53* in endometriosis and endometrial cancer (24, 25). In addition, two associated studies have revealed the link between *TP53* polymorphism and the risk of endometriosis (26, 27). Interestingly, *TP53* has been validated as a miR-125b target in several investigations by different methods, including reporter assay, western blot, and qPCR (23). However, the dysregulation of miR-125b and *TP53* between ectopic and eutopic endometrial samples has rarely been investigated (28).

Taken together, the relationship between endometriosis, cancer risk, miR-125b, and *TP53* encouraged us to compare the expression of both miR-125b and *TP53* in three kinds of samples with each other (Ec-p, Eu-p, and Normal). In addition, we used the bioinformatics evidence to investigate that if *TP53*, among all the potential miR-125b targets, has noticeable roles in cellular processes or not?

## 2. Materials and Methods

### PPI network construction 

In order to provide a comprehensive, genome-scale analysis of miR-125b regulatory networks based on its co-expressed targets; co-expression meta-analysis of miRNA Targets (CoMeTa) database was used (26). It is hypothesized that the targets of miR-125b are co-expressed with each other, and they belong to the same gene-regulatory network. In this database, three sequence-based prediction tools including miRanda, PicTar, and TargetScan are used. We have kept the top 100 ranks of co-expressed target genes. Then, STRING database v10.5 (functional protein association networks) was run to identify the protein interactions between them (29). In the next step, Cytoscape v3.6.1 was applied to visualize the PPI networks (30). Finally, the Molecular Complex Detection (MCODE), a Cytoscape app, was used to identify the module (31).

### Functional enrichment analysis

The Enrichr web server was applied for functional annotation of module members based on GO biological process and GO molecular function (32). P-value < 0.05 was considered to be significant enrichment.

### Sample collection

In this case-control study, the eutopic and ectopic samples were collected from women with regular menstrual cycles in the late luteal phase and referred to the Shahid Sadoughi Hospital, Yazd, Iran. Case samples were obtained from endometriosis patients who underwent laparoscopic surgery. Two biopsies were randomly removed from the endometriosis tissues (Ec-p, N = 20), and the endometrium inside the uterus (Eu-p, N = 20). The diagnosis of endometriosis in case samples was confirmed by histology in all women with the disease. The control subjects (Normal, N = 20) consisted of those women who had no evidence of endometriosis but were referred to the hospital for reasons such as fallopian tube blockage and pelvic pain and in whom no evidence of pelvic inflammation or any reproductive system diseases were found after laparoscopy. The mean age in the case group was 32 ± 2 yr (20-45 yr) and in the control group was 36 ± 2 yr (22-43 yr). Women aged 20-45 yr with regular menstrual cycles (28 to 32 days) were included in the study. All samples were stored in RNase free microtubes containing RNAlater${}^{\mathrm{TM}}$ Stabilization Solution (Thermo Fisher Scientific) at -80°C for later use. The exclusion criteria, on the other hand, were hormone therapy usage within 3 months before surgery, pregnancy, cancer, and contamination with common DNA viruses and human papilloma virus as well. Cancer patients were excluded because we wanted to find the relation with endometriosis as a cancer-like features and the genes under study and not the cancer itself.

### RNA extraction and cDNA synthesis

The total RNA was extracted from samples using Trizol reagent (Invitrogen) according to the manufacturer's instructions. The ratio of absorbance at 260 nm and 280 nm (A260/280) was used to assess RNA purity and quantity by spectrophotometer. Revert Aid First Strand cDNA Synthesis Kit (Fermentase, USA) was applied to synthesize cDNA of mRNA. Additionally, the Bon-Mir RT kit (Bonyakhteh, Tehran, Iran) was used to synthesize cDNA of miRNA based on the manufacturer's instructions. The resultant cDNA mixtures were stored at -20°C.

### Gene expression study 

Real-time PCR was performed using the commercial master mix (Takara, Japan). The sequences of primers were: SNORD forward 5'-ATCACTGTAAAACCGTTCCA-3', miR-125b forward primer as well as universal reverse primers for both miR-125b and SNORD were obtained from Bonyakhteh (Bonyakhteh, Tehran, Iran), *TP53* forward 5'-GCTCAGATAGCGATGGTCTGG-3', *TP53* reverse 5'-CTGTCATCCAAATACTCCACACG-3', GAPDH forward 5'-CTCATTTCCTGGTATGACAACGA-3', GAPDH reverse 5'-TCTTCCTCTTGTGCTCTTGCTG-3'. Moreover, the miR-125b and *TP53* expression levels were normalized by SNORD and GAPDH expression, respectively. Reaction mixture included 2 μl cDNA templates, 0.5 μl forward and reverse primers, 7 μl ribonuclease-free water, and 10 μl commercial master mix for a final reaction volume of 20 μl. The thermal cycling conditions were initiated with denaturation at 95°C for 10 min, and then 40 cycles at 95°C for 15 sec, and annealing at 60°C for 20 sec. For miR-125b detection, the real-time PCR profile was as described earlier, except for a melting temperature of 57°C. Each reaction was performed in duplicate, and expression levels were evaluated using two (-Δct).

### Cancer-related pathway analysis of miR-125b

DIANA miRPath was run to miR-125b pathway analysis based on experimentally validated miRNA interactions derived from TarBase database (33). Cancer-related KEGG pathways were extracted and visualized by Cytoscape software.

### Ethical consideration 

This study was approved by the ethics committee of Yazd Reproductive Sciences Institute, Shahid Sadoughi University of Medical Science, Yazd, Iran (code: IR.SSU.MEDICIN.REC.1396.151) and written informed consents were obtained from all individuals.

### Statistical analysis 

Statistical calculations were performed using the SPSS (Statistical Package for the Social Sciences, version 21.0, SPSS Inc, Chicago, Illinois, USA) software. Data were presented with mean and 95% CI. Kruskal-Wallis ANOVA was applied to analyze miR-125b or *TP53* expression among case-eutopic, case-ectopic, and control tissues. Mann-Whitney U-test was used to analyze expression between two groups. Data were considered significant when p < 0.05 was obtained. The Pearson's correlation statistic was used to evaluate the linear correlation between *TP53* and miR-125b expression. The linear regression model was applied to evaluate the linear effect of miR-125b expression on *TP53* expression.

## 3. Results

### PPI network of predicted miR-125b targets 

CoMeTa data were used to provide miR-125b regulatory network based on its co-expressed targets. PPI network has been constructed for potential miR-125b targets, which are co-expressed by STRING database. STRING outputs were further visualized using Cytoscape (Figure 1a). *TP53* with the highest degree (the number of incoming and outgoing edges) and between centrality (a measure of centrality in a graph based on shortest paths) was selected as a hub gene. After that, the top module was extracted by MCODE. The results showed that *TP53* gene is included in the top module (Figure 1b).

### Functional enrichment analysis of module 

All the module members were uploaded into the online Enrichr database to investigate the GO categories. The results revealed that module members are significantly enriched in cell proliferation, apoptosis, cell migration, and cell signaling (Table I).

### miR125b and *TP53* expression in endometriosis 

**Figure 2:**
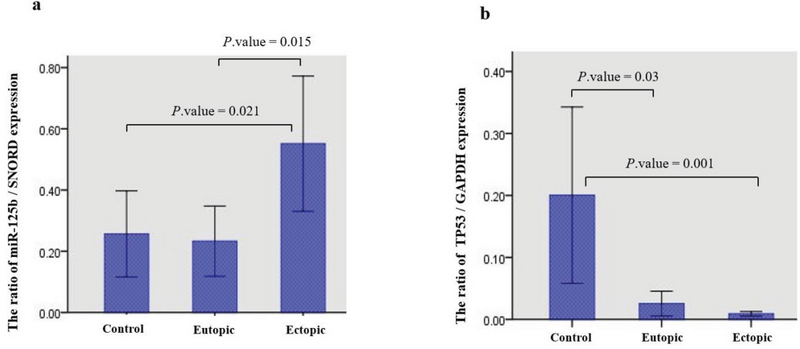
Differences in expression among Eu-p, Ec-p, and Normal control samples; a) miR-125b, SNORD. b) *TP53* GAPDH (Error Bars: 95% CI).

The analysis of miR125b and *TP53* expression in Eu-p and Ec-p as well as Normal tissues by Kruskal-Wallis test indicated that both miR125b (p = 0.024) and *TP53* (p = 0.003) expression were significantly different between cases (Eu-p and Ec-p) and Normal control samples. miR-125b in the Ec-p and *TP53* in the Normal control tissues had the highest expression levels. In addition, Mann-Whitney test outputs revealed that miR-125b expression was significantly different between the Eu-p and Ec-p tissues (p = 0.015) as well as between the Ec-p and Normal control tissues (p = 0.021). Concerning *TP53* expression, Mann-Whitney test indicated that its expression was significantly different between the Eu-p and Normal control tissues (P-value = 0.03) as well as between the Ec-p and Normal control tissues (p = 0.001) (Figure 2).

### Correlation between *TP53* and miR-125b expression 

**Figure 3:**
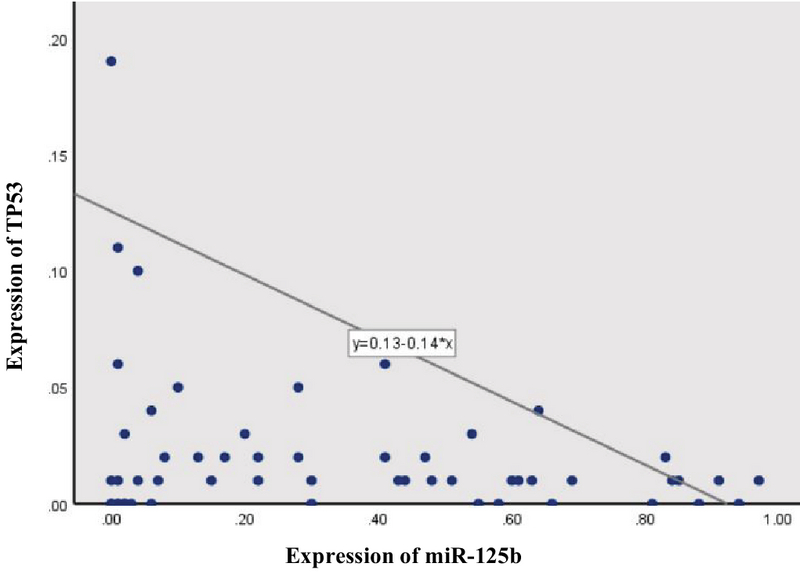
Association between *TP53* and miR-125b expression in all samples (n = 60, r = -0.26. p = 0.04). Regression equation by linear regression analysis.

A negative correlation was seen between the expression of the two targeted genes (r = -0.2, p = 0.04) among all samples (Figure 3). The linear regression model revealed that for every 1 unit additional miR-125b expression in samples, we would expect to see a 0.14-unit decreseas in the *TP53* expression (Figure 2).

### Cancer-related KEGG pathways of miR-125b

DIANA miRPath database revealed that miR-125b has several experimentally validated target genes, which are involved in cancer-related KEGG pathways, such as endometrial and ovarian cancers. Among the miR-125b-validated targets, *TP53* is associated with more cancer-related pathways (Figure 3).

**Table 1:** Functional enrichment analysis of module members according to the online Enrichr database based on GO biological process and GO molecular function


**Ontologies**		**Gene ontology term**	**P-value**
**GO biological process 2018**			
	**Mammary gland development**	0030879	0.00004
	**Positive regulation of cell migration involved in sprouting angiogenesis**	0090050	0.000001
	**ERBB2 signaling pathway**	0038128	0.00002
	**Negative regulation of programmed cell death**	0043069	0.00001
	**Negative regulation of apoptotic process**	0043066	0.00001
	**Regulation of cell population proliferation**	0042127	0.0001
	**Positive regulation of cell migration by vascular endothelial growth factor signaling pathway**	0038089	0.002
	**Positive regulation of vascular endothelial cell proliferation**	1905564	0.003
**GO molecular function 2018**			
	**Growth factor receptor binding**	0070851	0.00003
	**Epidermal growth factor receptor binding**	0005154	0.0003
	**Mitogen-activated protein kinase kinase binding**	0031434	0.0003
	**Vascular endothelial growth factor receptor binding**	0005172	0.003
	**Vascular endothelial growth factor receptor 2 binding**	0043184	0.002
Fisher's exact test was used. GO: Gene ontology ERBB2: Erb-B2 Receptor Tyrosine Kinase 2			

## 4. Discussion

Endometriosis is a common chronic disease and affects about 10-15% of women of reproductive age. This disease is characterized by the growth of endometriotic tissue outside the uterine. Although endometriosis is generally considered as a benign condition, it shows a risk for transformation and becoming cancerous (34). miRNAs, as a non-coding regulatory RNAs, are involved in post-transcriptional gene regulation. Dysregulation of miRNAs in endometriosis and cancers have been reported in the previous studies (35, 36). Hawkins and colleagues have shown several upregulated and downregulated miRNAs in endometriomas compared with the normal endometrium (37). Also, Ohlsson Teague and co-workers have found dysregulated miRNAs between pairs of ectopic and eutopic endometrial tissues (19). These investigations indicate the roles of miRNAs in the development of endometriosis.

Phenotypic resemblance between endometriotic and malignant cells such as uncontrolled cell growth and decreased apoptosis indicates their common molecular features. In addition, it is suggested that miRNAs and their targets can be considered as effectors in both endometriosis and endometriosis-associated cancers. The identification of dysregulated miRNAs and their targets in the eutopic and ectopic endometrium is an essential step toward understanding molecular links between endometriosis and cancers. Moreover, this information can be useful for developing therapeutic strategies. In the current study, in the first step, we selected miR-125b and investigated its potential targets with bioinformatic approach. In the previous studies, miR-125b had shown a significant upregulation in both endometriotic tissues and serum samples of women with endometriosis (19, 20). In addition, the role of this microRNA as an oncomiR have been reported in cancers. Zhou and co-authors indicated that mir-125b acts as an apoptosis suppressor in breast cancers (38). Furthermore, Bousquet and colleagues suggested that miR-125b confers a proliferative advantage to the leukemic cells (39). Similarly, Xia *et al* demonstrated that overexpression of miR-125b promotes human glioma cell proliferation (40).

Here, miR-125b regulatory network was investigated and PPI network was constructed based on its co-expressed potential targets. These analyses indicate that *TP53* is a remarkable potential target for miR-125b. Moreover, the top module in the PPI network includes *TP53* protein (Figure 1). Module-based functional pathway enrichment analysis has revealed roles of *TP53* and other members of module in different cancer-related pathways such as regulations of cell migration, of vascular endothelial cell proliferation, and of apoptotic process (Table I). As far as the association between endometriosis and cancer is concerned, our bioinformatic analyses point out that *TP53* is a potential appropriate target for miR-125b. Interestingly, the interaction between miR-125b and *TP53* miRNA has been validated in human neuroblastoma, myeloid, and cardiac fibroblasts cells (23, 41, 42). It is shown that the loss of miR-125b during zebrafish embryogenesis leads to aberrant apoptosis due to overexpression of *TP53*. Jiang and colleagues proposed that miR-125b inhibits *TP53* network activity by regulating the dose of both proliferative and apoptotic regulators (43). In the present study, the expression of miR-125b and *TP53* in eutopic and ectopic endometrium of women with endometriosis as well as normal control tissues were explored. Our results revealed a negative correlation between miR-125b and *TP53* expression in the three types of samples. These findings suggest that miR-125b regulates *TP53* expression in endometrium. The ectopic tissues showed the highest expression of miR-125b and the lowest expression of *TP53*.

Furthermore, several studies have reported inhibitory roles of *TP53* in cell invasion and metastasis (44, 45). This finding can explain the characteristics of ectopic endometrium such as greater ability of migration and invasion compared with eutopic endometrium (46). Our data demonstrated that expression of both miR-125b and *TP53* is significantly different between ectopic endometrium and normal control samples. Moreover, while there was no overexpression of miR-125b in eutopic samples, it significantly showed low expression of *TP53* compared with normal samples. This could be due to an overexpression of miR-125b in Ec-P samples and its effect on all of the patient's tissues including Eu-p tissues. Consistently, Allavena and co-workers showed that the expression of *TP53* decreases in the ectopic endometrium of patients compared with eutopic and normal samples (25).

Our results, consistent with previous studies, revealed that *TP53* expression decreases in benign endometriotic cysts compared with normal controls. This observation can suggest that alterations in the *TP53* expression, as a tumor suppressor gene, may be involved in the same molecular features between endometriosis and cancers. Our results, according to previous studies, demonstrates the existence of negative correlation between the expression of miR-125b and *TP53* in both the case and control samples (Figure 3).

**Figure 4:**
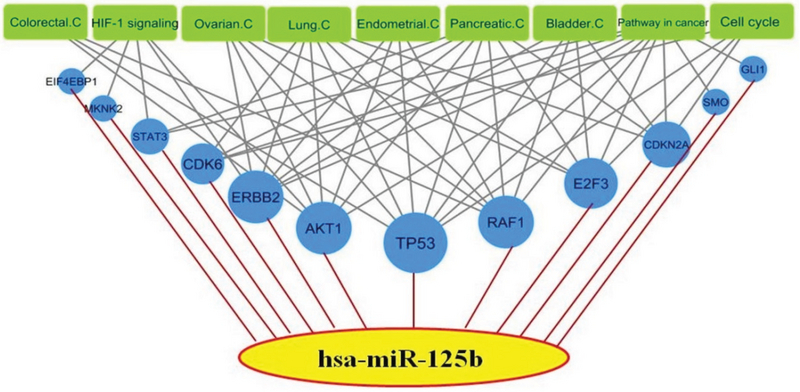
Cancer-related KEGG pathways of miR-125b. The blue nodes represent experimentally validated targets of miR-125b. The green nodes represent cancer-related KEGG pathways. The size of blue node is proportional to the node degree. Data were obtained from DIANA-miRPath database.

Finally, in order to investigate the potential roles of miR-125b in endometriosis-associated cancers, we constructed cancer-related KEGG pathways of validated targets of miR-125b. Among the miR-125b-validated targets, such as *TP53*, *STAT3*, *E2F3*, *CDKN2A*, *AKT1,* and *ERBB2*, *TP53* shows association with more cancer-related pathways (Figure 4). A combination of this analysis and our experimental data indicates multiple roles of miR-125b, which may underlie endometriosis-associated cancers. However, since both tumor suppressor genes and oncogenes are seen among validated targets of miR-125b, it is proposed that miR-125b has an opposing function as an oncogene and a tumor suppressor in different cell contexts. Further studies are necessary for investigating the association between miR125b expression and developing endometriosis-associated cancers in women with endometriosis.

## 5. Conclusion

In conclusion, the negative correlation between miR-125b and *TP53* as well as the noticeable decreased expression of *TP53* in both eutopic and ectopic samples compared with Normal controls may be interpreted in roles of miR-125b/*TP53* axis in the pathogenesis of endometriosis. In addition, these finding and bioinformatics analyses imply a possible role of miR-125b in the connection between endometriosis and malignant transformation.

##  Conflict of Interest

The authors declare that there is no conflict of interest that could be perceived as prejudicial to the impartiality of the reported research.
